# Sorghum Panicle Detection and Counting Using Unmanned Aerial System Images and Deep Learning

**DOI:** 10.3389/fpls.2020.534853

**Published:** 2020-09-02

**Authors:** Zhe Lin, Wenxuan Guo

**Affiliations:** ^1^ Department of Plant and Soil Science, Texas Tech University, Lubbock, TX, United States; ^2^ Department of Soil and Crop Sciences, Texas A&M AgriLife Research, Lubbock, TX, United States

**Keywords:** deep learning, computer vision, sorghum panicle, unmanned aerial systems, convolutional neural networks, python, TensorFlow, image segmentation

## Abstract

Machine learning and computer vision technologies based on high-resolution imagery acquired using unmanned aerial systems (UAS) provide a potential for accurate and efficient high-throughput plant phenotyping. In this study, we developed a sorghum panicle detection and counting pipeline using UAS images based on an integration of image segmentation and a convolutional neural networks (CNN) model. A UAS with an RGB camera was used to acquire images (2.7 mm resolution) at 10-m height in a research field with 120 small plots. A set of 1,000 images were randomly selected, and a mask was developed for each by manually delineating sorghum panicles. These images and their corresponding masks were randomly divided into 10 training datasets, each with a different number of images and masks, ranging from 100 to 1,000 with an interval of 100. A U-Net CNN model was built using these training datasets. The sorghum panicles were detected and counted by a predicted mask through the algorithm. The algorithm was implemented using Python with the Tensorflow library for the deep learning procedure and the OpenCV library for the process of sorghum panicle counting. Results showed the accuracy had a general increasing trend with the number of training images. The algorithm performed the best with 1,000 training images, with an accuracy of 95.5% and a root mean square error (RMSE) of 2.5. The results indicate that the integration of image segmentation and the U-Net CNN model is an accurate and robust method for sorghum panicle counting and offers an opportunity for enhanced sorghum breeding efficiency and accurate yield estimation.

## Introduction

Sorghum (*Sorghum bicolor* L. Moench) is the fifth top cereal crop in the world, which provides nutrition to humans and livestock, particularly in warm and arid climates (FAO, 1999). Sorghum is one of the most eﬃcient crops in the conversion of solar energy and the use of water. It has numerous varieties, including grain sorghums used for human food, and forage sorghum for livestock hay and fodder (Dahlberg et al., 2015). By measuring the plant population and the weight per panicle, growers can estimate the potential final grain yield (Norman et al., 1995). However, it is challenging to determine plant population by manually counting sorghum panicles, especially for large fields. Traditional counting methods for yield estimation are mainly focused on hand-sampling in the field, which is tedious, time-consuming, labor-intensive, and prone to human errors. Therefore, it is critical to develop alternative methods to efficiently and accurately count sorghum panicles for determining population and estimating yield.

Technological innovations in platforms and advanced sensors such as unmanned aerial systems (UAS) and efficient image processing capabilities provide opportunities to automate high-throughput plant phenotyping through computer vision. UAS imaging has been widely used in plant phenotyping and precision agriculture-related research. Many low-cost sensors onboard UAS can provide aerial images with centimeter-level spatial resolutions. Further, UAS allows for more flexibility in image acquisition in terms of flight height, flight area, and weather conditions. Different sensors onboard the UAS offer various ways for researchers and growers to characterize plant attributes at different scales. As a result, UAS has become a useful platform for crop growers and researchers to acquire aerial images with high spatial and temporal resolutions for quantifying within-field variations (Gómez-Candón et al., 2014). For example, RGB (red, green, and blue bands) cameras, multispectral and thermal sensors were applied to estimate LAI (Hunt et al., 2010; Verger et al., 2014), biomass (Hunt et al., 2005; Bendig et al., 2015), water stress (Gago et al., 2015; Ballester et al., 2018), plant height (Bendig et al., 2015; Díaz-Varela et al., 2015), plant density (Jin et al., 2017; Liu et al., 2017), plant counts (Chen et al., 2017b; Gnädinger and Schmidhalter, 2017; Guo et al., 2018; Olsen et al., 2018; Oh et al., 2019), plant and soil temperature (Gómez-Candón et al., 2016; Zhang et al., 2018), and plant nitrogen status (Hunt et al., 2005; Tokekar et al., 2016). Yang et al. (2017) provided a review on how UAS remote sensing and multiple sensors were applied in field-based plant phenotyping.

Image segmentation is commonly the first step to extract information of targets from an image by separating a set of pixels containing the objects of interest (Mochida et al., 2018). The application of image segmentation for plant phenotyping is typically implemented at small scales because the input requires detailed information with accurate labels, which is time-consuming and labor-intensive. Machine learning, together with computer vision, offer opportunities for high-throughput plant phenotyping in recent years. Machine learning can be broadly defined as computational methods to make accurate predictions or improve performance using experience (Mohri et al., 2018). Deep learning refers to a class of machine learning techniques that leverage multiple layers of non-linear information processing for unsupervised or supervised feature extraction and transformation, and for classification and pattern analysis (Deng et al., 2014). Deep learning algorithms learn high-level features in an incremental way, which eliminates the need for feature identification and extraction (LeCun et al., 2015). The deep networks have the capacity to learn complex models that involve crop phenotypic attributes. A variety of vision-based algorithms have been proven effective with high accuracy in plant phenotyping, such as crop or leaf recognition (Sankaran et al., 2015; Gómez-Candón et al., 2016; Sladojevic et al., 2016), disease detection (Barbedo, 2014; Pérez-Ortiz et al., 2015; Too et al., 2019), crop classification (Makantasis et al., 2015; Dyrmann et al., 2016; Kussul et al., 2017), and crop or fruit counting (Pape and Klukas, 2015; Chen et al., 2017b; Qureshi et al., 2017; Guo et al., 2018; Hasan et al., 2018; Olsen et al., 2018; Ubbens et al., 2018; Madec et al., 2019; Oh et al., 2019; Xiong et al., 2019). In recent years, traditional machine learning and deep learning algorithms were used on image segmentation, especially in the areas of biomedical and object detection. For example, Chen et al. (2017a) developed the Deeplab system and Fully Convolutional Network for semantic image segmentation. Ronneberger et al. (2015) used a U-Net convolutional neural networks (CNN) algorithm with limited training images for the segmentation of neuronal structures in electron microscopic images. Few studies integrated image segmentation in traditional machine learning or deep learning models for plant phenotyping applications. Islam et al. (2017) detected potato diseases on individual leaves using image segmentation and the multiclass support vector machine. Wu et al. (2019) combined image segmentation with VGG-16 CNN on automatic counting of rice seedlings from UAS images. Traditional machine learning and deep learning architectures have been applied to sorghum panicle detection and counting. Guo et al. (2018) used a two-step, decision-tree-based pixel segmentation model (DTSM), and Support Vector Machine (SVM) method with the Classification Learner in sorghum panicle detection. Olsen et al. (2018) developed a machine learning algorithm using image annotation to detect and count sorghum panicles with a mean absolute error of 2.66. Ghosal et al. (2019) proposed a weakly supervised semi-trained CNN model using UAS images for sorghum panicle detection and rough localization. Therefore, image segmentation, together with machine learning, has the potential to detect sorghum panicles and estimate the panicle shape, which can further improve the accuracy of yield prediction.

For effective deep learning algorithms in agricultural applications, model selection and feature definition are critical, which heavily rely on specialized knowledge in both plant phenotyping and computer science (Singh et al., 2016). Environmental factors such as cloud and windy weather can significantly impact the quality of agricultural images (Ghosal et al., 2019). In addition, plant phenotyping based on UAS images is also sensitive to plant genotypes, sensor-target angles, overlap among leaves and panicles, panicle damages, and field conditions. As a result, a large number of training images are required to accommodate various environmental conditions to obtain robust and accurate machine learning algorithms for plant phenotyping tasks. However, building a large number of training samples requires a long time and heavy labor. As a result, datasets of crop images are not yet available on a large scale due to the expenses involved in collecting and preparing the corresponding training data. Therefore, it is critical to develop algorithms that determine the appropriate number of images to meet the requirement of accurate plant phenotyping, such as sorghum panicle counting. The objectives of this study were to 1) develop a deep learning CNN image segmentation algorithm to detect and quantify sorghum panicles; 2) evaluate the performance of this algorithm with respect to the number of training images.

## Materials and Methods

### Experimental Sites

This study was conducted in a research field (33° 35’ 50.53’’ N, 101° 54’ 27.30’’ W) in Lubbock, Texas, in 2018. The climate in this region is semiarid, with an average annual rainfall of 487 mm, mostly falling between May and September, frequently as the result of convective thunderstorms (US Climate Data, 2019). Three sorghum varieties, including NK180, XM217, and KS585 (S&W Seed Company, Sacramento, CA) with two seed populations of 120 and 180, were planted on May 26, 2018. In total, there were 120 plots, each of 6 m long and eight rows wide in an east-west direction. A 1.5-m alley was arranged between plots. The NK180 is a drought-tolerant, bird resistant, and early-maturity variety. The NK180 has a whitish color and a relatively large sorghum panicle. The average measured panicle length for this variety in this study was 22 cm. The XM217 has a red color and a relatively small sorghum panicle. The average panicle length was 13 cm. The KS585 is a drought-tolerant, medium height, and medium-maturity variety. The KS585 has a light brown color, which is close to the soil color, and a relatively small sorghum panicle. The average panicle length was 14 cm. A subsurface drip irrigation system was used for irrigation in this field during the growing season.

### UAS Image Collection

A DJI Phantom 4 Pro UAS (DJI, Shenzhen, China) with a 4K RGB camera was applied in image acquisition. The UAS has a 2-axis gimbal that can maintain the orientation of the camera independently from the movement. The UAS is controlled with a 2.4 GHz frequency bidirectional transmission that receives data of the battery voltage, Global Positioning System (GPS) reception, the distance, and the height differences from the home point. The maximum flight duration of the UAS is about 30 min. The flight plan was created using the Pix4Dcapture software (Pix4D S.A., Switzerland). The flight plan included 80% front overlap and 80% side overlap. The angle of the camera was set at 90 degrees to the land surface during flight. The UAS was flying at an altitude of 10 m at 2.7 m s^-1^ speed. The spatial resolution was 2.7 mm for 10 m altitude. Two image datasets were acquired on August 24 and September 10, 2018. All image acquisitions were completed under sunny conditions with light to moderate wind around local solar noon. Raw images were stitched into a whole image using the Pix4DMapper software (Pix4D S.A., Switzerland).

**Figure 1:**
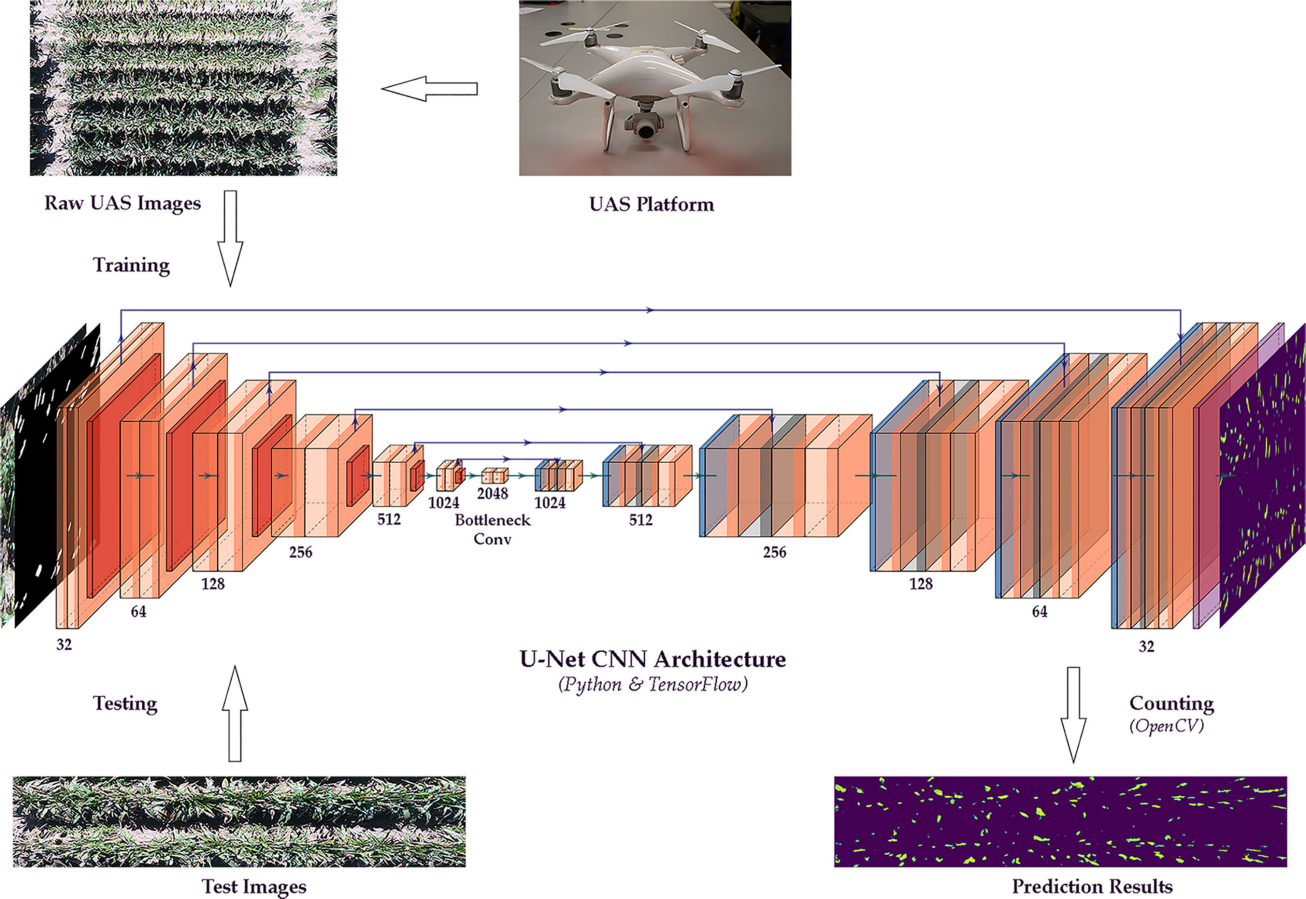
Flow chart of a sorghum panicle detection and counting algorithm using a U-net Convolutional Neural Networks model on unmanned aerial system images.

This study applied an integrated method of image segmentation and deep learning for sorghum panicle detection and counting. **Figure 1** shows the steps of the algorithm for sorghum panicle detection and counting. The U-Net CNN (Ronneberger et al., 2015) was adopted as the deep learning framework to train and test the image data.

### Preparing Training Images and Masks

**Figure 2:**
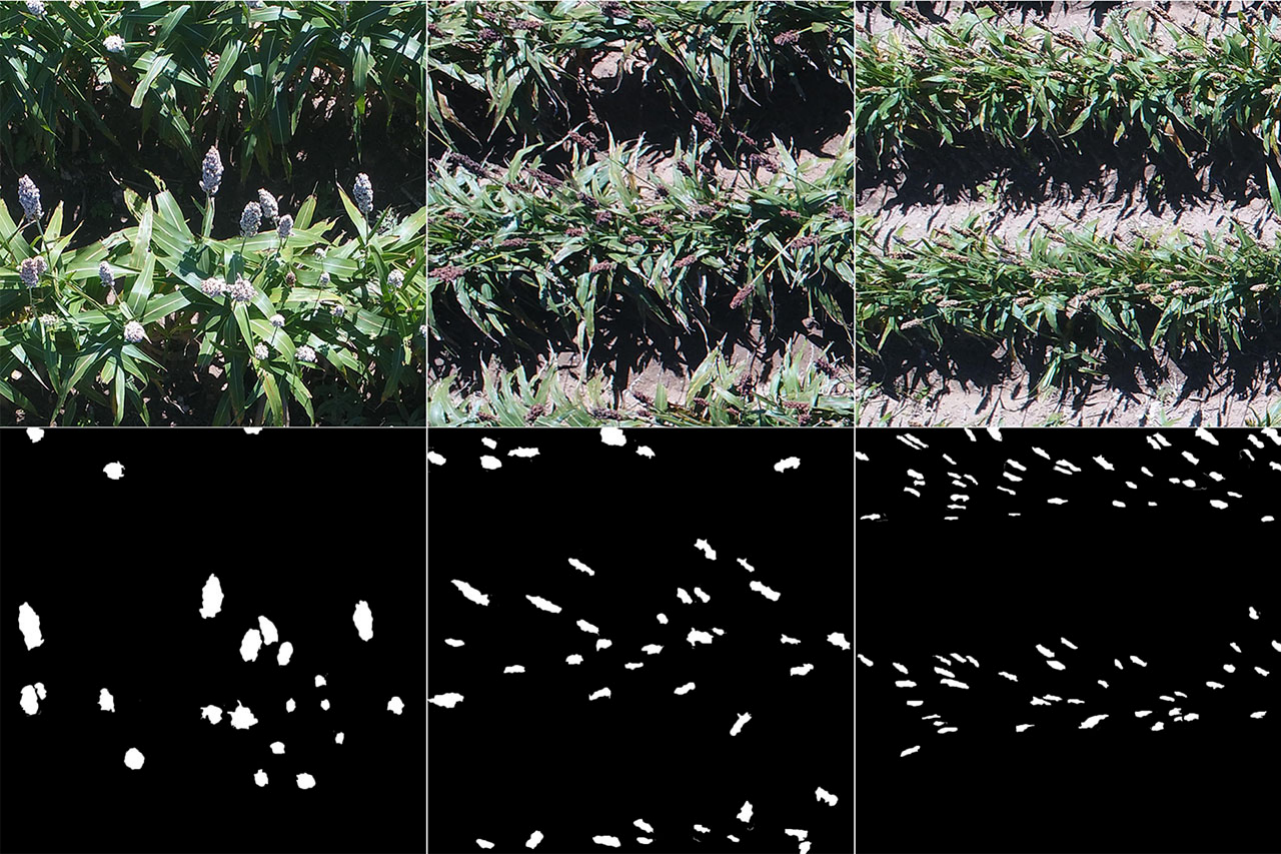
Examples of training images (Top) and corresponding masks (Bottom) for a sorghum panicle detection and counting algorithm using a Convolutional Neural Networks model on unmanned aerial system images (Left to right: NK180, XM217, and KS585).

The training images were prepared by randomly cropping the raw UAS images using the Microsoft Paint 3D software (Microsoft Corporation, Redmond, WA). To accurately separate sorghum panicles from other objects in the image, a segmentation mask for each training image was created by encircling the sorghum panicle pixels using the Adobe Photoshop CC software (Adobe Systems Inc., San Jose, CA). Specifically, for each training image of 1024 x 1024 pixels, pixels were divided into two classes, the sorghum panicle class and the non-panicle class. In the mask, the pixels containing sorghum panicles were digitized as white and assigned a value of 1, while the other pixels were set black and assigned the value of 0 (**Figure 2**). These mask images were saved separately to ensure that each mask matched its corresponding training image when running the U-Net CNN model. The full training dataset contained 1,000 images. To test the model performance as a function of the number of training images, a series of 10 randomly selected sub-datasets, ranging from 100 to 1,000 with an interval of 100 images (i.e., 100, 200, …, 1000 images), were generated from the full training dataset. Each sub-dataset was used to train a model and tested for the accuracy of the panicle count for the corresponding number of training images.

### U-Net Convolutional Neural Networks

The general procedure of the U-Net CNN in this study is described as follows. The U-Net architecture consists of three sections: the contraction, the bottleneck and the expansion. In this study, there were six blocks in the contraction and the expansion sections. The kernel size was 3 x 3 and the strides were 1 x 1 in the contraction section. In the expansion section, the kernel size was 2 x 2 and the strides were 2 x 2. No padding was applied in either section. In the contraction section, each block contained two convolution layers, followed by a down-sampling layer. Once every pixel was processed after the convolution layers, the result was saved into a new feature map in the same arrangement as the input image. The down-sampling layer was used to reduce the feature map dimension, so only the most essential bits of the feature map were kept. The reduced feature map was then utilized as an input to the next contraction block. The spatial dimensions of the feature maps were halved and the number of feature maps was doubled repeatedly through the down-sampling layer (Guan et al., 2019; Weng et al., 2019). The bottleneck layer, which contained two convolution layers but without max pooling, mediated the contraction section and the expansion layer. The data at the bottleneck had the spatial dimension of 32 x 32 with 2048 feature maps. In the expansion section, the block contained two convolution layers followed by an up-sampling layer. After each up-sampling layer, the number of feature maps was halved and the spatial dimensions of the feature maps were doubled to maintain the whole architecture symmetry. In the meantime, the input from the corresponding contraction block was appended to the feature maps. After running all the expansion blocks, the final output feature map with the same spatial dimension as the original input image included the sorghum panicle class and the non-panicle class.

### Segmentation Model Training and Validation

For each training dataset, 90% of the images were set as training, and the rest 10% was used as validation for the training models. For example, in a 500-image dataset, 450 images were trained through the model, and the rest 50 images were used as validation. Before training the segmentation model, all training images and masks went through the image augmentation processes. The hue of each RGB training image was adjusted by a factor of 0.1. Both the training images and corresponding masks were flipped horizontally along the central axis with a 0.5 probability. The training images and corresponding masks were randomly shifted either horizontally or vertically. Finally, both training images and the corresponding masks were rescaled by a factor of 1/1023. The purpose of image augmentation was to increase the amount of training data by applying some transformations to the original training images. This helps the model to generalize better to unseen data and prevent overfitting (Wang and Perez, 2017; Frid-Adar et al., 2018; Mikołajczyk and Grochowski, 2018). After the image augmentation process, a two-channel segmentation model was generated from these training images and masks using the U-Net algorithm.

The pixel-wise cross-entropy loss function was used to evaluate the training models of the U-Net CNN algorithm using the 10% validation images in the training datasets. The cross-entropy loss is commonly used as a loss function for training in deep learning networks, especially in image segmentation tasks (Ronneberger et al., 2015; Sudre et al., 2017; Martins and Zaglia, 2019). Cross-entropy loss measures the probability difference between the observed and the predicted values in a classification model (Buja et al., 2005). The cross-entropy loss (CE) for the binary classification in this study is defined as,

(1) $$CE=-(y_{i}log(p_{i})+(1-y_{i})log(1-p_{i}))$$

where *y_i_* represents the labeled value for that sample in the mask, and ***p_i_*** represents the predicted probability being the sorghum panicle in the output feature maps.

### Counting and Evaluations

A test dataset containing 120 images was selected from the fully stitched image for accuracy assessment. The images in the test dataset were different from the images in the training dataset. Each test image was corresponding to two rows of sorghum plants randomly selected from a plot. Sorghum panicles in each test image were manually counted, and the number of sorghum panicles in these test images varied from 95 to 188. The size of each test image was 3800 x 1280. We found out that it was difficult for the U-Net CNN model to process the high-resolution test images directly. In this case, we horizontally split each test image into four non-overlapped subtest images. Before running the model on the test images, the subtest images for each test image were resized to dimensions of 1024 x 1024 pixels. Then the test images were run through the trained segmentation model to perform the panicle detection. Each sorghum panicle detected was treated as a contour using the *findContours* function of the OpenCV library in the prediction output feature map. Our initial assessment indicated that contours with less than six pixels were mainly noise related and not classified as panicles. A bounding polygon was applied around each panicle contour using the *drawContours* function for each subtest image. Therefore, the number of bounding polygons represented the number of predicted sorghum panicles in each subtest image. The summation of sorghum panicles of the four subtest images equaled the total number of sorghum panicles in each test image.

The mean absolute error (MAE), mean absolute percentage error (MAPE), accuracy (ACC), coefficient of the determination (R^2^), and the root mean squared error (RMSE) were used as evaluation metrics to assess the performance of the sorghum panicle counting algorithm.

(2) $$MAE=\frac{1}{n}\sum_{1}^{n}\left|m_{i}-c_{i}\right|$$

(3) $$MAPE=\frac{1}{n}\sum_{1}^{n}\left|\frac{m_{i}-c_{i}}{m_{i}}\right|$$

(4) $$ACC=(1-\frac{1}{n}\sum_{1}^{n}\frac{\left|m_{i}+c_{i}\right|}{m_{i}})\times 100\%$$

(5) $$R^{2}=1-\frac{\sum_{1}^{n}{\left(m_{i}-c_{i}\right)}^{2}}{\sum_{1}^{n}{\left(m_{i}-\bar{m_{i}}\right)}^{2}}$$

(6) $$RMSE=\sqrt{\frac{\sum_{1}^{n}{\left(m_{i}-c_{i}\right)}^{2}}{n}}$$

where m_i_, ${\bar{m}}_{i}$, and c_i_ represent the manually counted sorghum panicles for the i^th^ image, the mean manual counts, and the predicted count for the i^th^ image, respectively. n is the number of test images.

### Hardware and Libraries Used

The algorithm was implemented using the Python programming language (Python Software Foundation, 1995). The model was trained on a computer with 192 GB of memory at the Texas Tech University High Performance Computing Center (HPCC). Training, evaluation, and testing were performed using the Keras (Chollet, 2015) high-level neural networks application programming interface (API), running on top of the TensorFlow package (Abadi et al., 2016). The model in this study was trained using the Adam (Kingma and Ba, 2015) optimizer with a learning rate of 0.001. Fifteen epochs were performed in the training process. The number of epochs was determined based on the training image size, training required time, and the overall performance of the model. In this study, the cross-entropy loss value did not decrease significantly beyond 15 epochs. The OpenCV-Python library (Bradski, 2000) was used in model testing.

## Results

### Training Model and the Number of Training Images

**Table 1** shows the overall decreasing trend of the cross-entropy loss with the number of training images, indicating an increasing accuracy in model performance with the number of training images. The value of cross-entropy loss did not decrease rapidly from 100 to 500 training images. On the other hand, from 600 training images, every 100 more training images resulted in a decrease of more than 0.10 in cross-entropy loss. The value of cross-entropy loss and the trend indicated that there could be potential to improve the performance of the segmentation model by increasing the number of training images. However, due to the restriction of the training period in our HPCC and the CPU memory, the value of the cross-entropy loss used here was based on 15 epochs of training for all different numbers of training images. Many studies have shown that the cross-entropy loss value could be close to 0 with a large number of training epochs (Alom et al., 2018; Zhang and Sabuncu, 2018). However, with the number of training epochs, the results of cross-entropy values showed a clear negative trend as the number of training images increased.

**Table 1:** Cross-entropy loss values for 10 sets of training images for a sorghum panicle detection and counting algorithm using a U-Net Convolutional Neural Networks model on unmanned aerial system images.

**No. of Images**	**100**	**200**	**300**	**400**	**500**	**600**	**700**	**800**	**900**	**1000**
Cross-entropy loss	0.71	0.66	0.63	0.59	0.53	0.50	0.39	0.34	0.20	0.11

### Sorghum Panicle Counting Performance and the Number of Training Images

**Figure 3:**
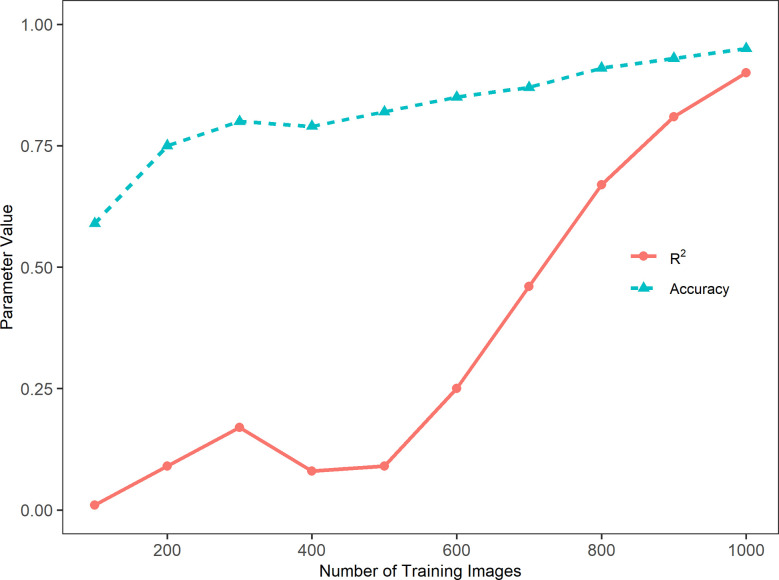
Trends of accuracy, Cross-Entropy Loss, and coefficient of determination (R^2^) with the number of training images in a sorghum panicle detection and counting algorithm using a Convolutional Neural Networks model on unmanned aerial system images.

**Figure 3** presents the accuracy and coefficient of determination (R^2^) of the model performance in relation to the number of training images. In general, the sorghum panicle count accuracy and R^2^ values increased with the number of training images. This trend, however, was not consistent for the cases with training images below 500. The accuracy was low at 59% with 100 training images, increased to 75% and 80% with 200 and 300 training images, respectively, but dropped slightly to 78% with 400 training images. Similarly, the R^2^ value was lowest at 0.01 with 100 training images, increased to 0.09 and 0.17 with 200 and 300 training images, but dropped to 0.08 with 400 images. For cases with more than 500 training images, the accuracies and R^2^ values consistently increased with the number of training images. With 1000 training images, the highest accuracy of 95.5%, and the highest R^2^ value of 0.90 were achieved. In addition, the rate of change in relation to the number of images for R^2^ was greater than that for the accuracy. From 500 to 1,000 images, the accuracy increased by 16.5% from 82% to 95.5%, while the R^2^ value increased by 900% from 0.09 to 0.90. This indicates the accuracy is a better parameter for evaluating this type of algorithm performance. In summary, the algorithm performance was not stable with less than 500 training images. With more than 500 training images, the algorithm performance steadily improved with respect to accuracy.

MAE, MAPE, and RMSE consistently decreased with the increasing number of training images (**Table 2**). These trends were not consistent with the trends for the accuracy and R^2^ values, which had fluctuations in the relation between the magnitude and number of training images. For a low number of training images, the MAE was relatively large; it was 53.1 for 100 training images and 35.2 for 200 training images. This value dropped to 6.3 for 1,000 images. MAPE was 0.41 for 100 training images and 0.25 for 200 training images, then it dropped to 0.05 for 1,000 training images. A similar trend was observed for the RMSE values. Its change, however, was not as extreme as those MAE values. RMSE was 7.3 for 100 training images, and it gradually dropped to 2.5 for 1,000 training images. Considering the range of sorghum panicles (95 to 188) in the test dataset, the MAE and RMSE values for 1,000 training images are within an acceptable range.

**Table 2:** Mean absolute error (MAE), mean absolute percentage error (MAPE), and root mean squared error (RMSE) for 10 sets of training images for a sorghum panicle detection and counting algorithm using a U-Net Convolutional Neural Networks model on unmanned aerial system images.

**No. of Images**	**100**	**200**	**300**	**400**	**500**	**600**	**700**	**800**	**900**	**1000**
MAE	53.1	35.2	28.1	27.2	23.7	19.6	16.9	11.4	9.9	6.3
MAPE	0.41	0.25	0.20	0.21	0.18	0.15	0.13	0.09	0.07	0.05
RMSE	7.3	5.9	5.3	5.2	4.9	4.4	4.1	3.4	3.2	2.5

**Figure 4:**
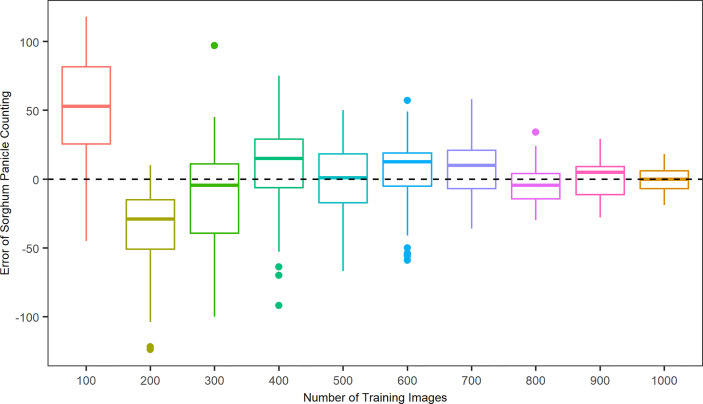
Distributions of counting errors between predicted and observed sorghum panicles for ten sets of training images using a Convolutional Neural Networks model on unmanned aerial system images.

To better evaluate the algorithm performance with respect to the patterns of over- and under-estimations, **Figure 4** shows the error, the difference in sorghum panicles between the model prediction and the manual count result, in relation to the number of training images. If the error is positive, then the algorithm overestimates sorghum panicles; otherwise, the algorithm underestimates. For the cases of 100, 400, 600, 700, and 900 training images, the results represented a mean overestimation of 50.1, 23.7, 20.1, 16.9, and 10.1 panicles, respectively. For 200, 300, and 800 training images, the results represented a mean underestimation of 35.2, 28.0, and 11.4 panicles, respectively. The mean errors were 3.9 and 2.6 for the cases of 500 and 100 images, respectively. However, the variance of prediction results for the 500 training images was larger than that for the 1000 training images. A key to the success of deep learning in object detection tasks is abundant training images. A larger number of training images results in better accuracy and performance (Kamnitsas et al., 2017; Aggarwal et al., 2019). Therefore, the accuracy and robustness of this algorithm increased with the number of training images, with 1,000 images providing the best performance.

**Figure 5:**
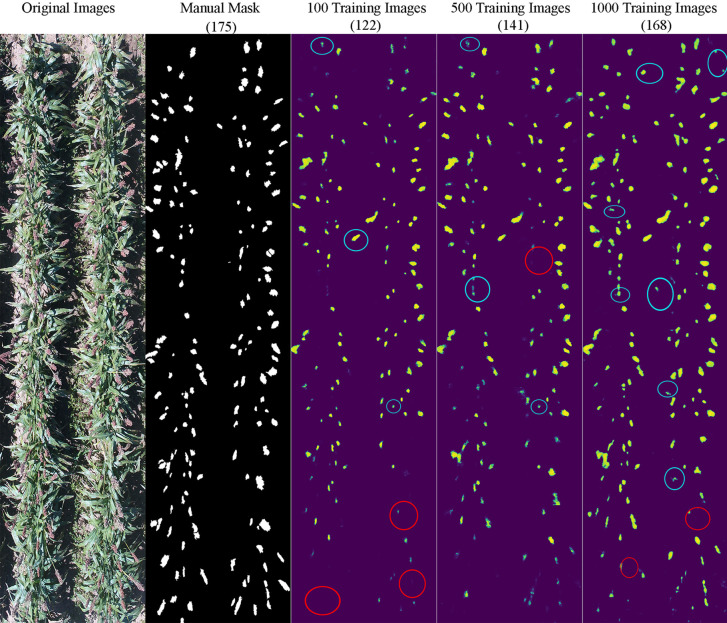
Sample results of sorghum panicle detection for Variety XM217 with 100, 500, and 1000 training images using a Convolutional Neural Networks model and UAS images. Red circles represent underestimation; blue circles represent overestimation compared to the manual masks.

It appeared that the counting accuracy was related to the soil background. **Figures 5** **–** **7** show the examples of sorghum panicle detection results for the three varieties with 100, 500, and 1,000 training images. For the case of 100 training images, the prediction was overestimated by 53 on average. This substantial overestimation was due to some soil pixels between plots being counted as sorghum panicles, especially for XM217 and KS585. The sorghum panicle colors of these two varieties were similar to the soil background. Therefore, with only 100 training images, the U-Net CNN algorithm was not able to distinguish the soil and sorghum panicles with similar colors. For the case of 500 training images, both overestimation and underestimation were observed. For XM217, 141 sorghum panicles were predicted compared to 175 values observed. For KS585, the predicted number of sorghum panicles was 163 compared with the observed number of 215. For NK180, 99 sorghum panicles were detected, while the observed was 114. Overlapping sorghum panicles and the misclassification between white soil background and sorghum panicles caused the overestimation and underestimation with 500 training images.

**Figure 6:**
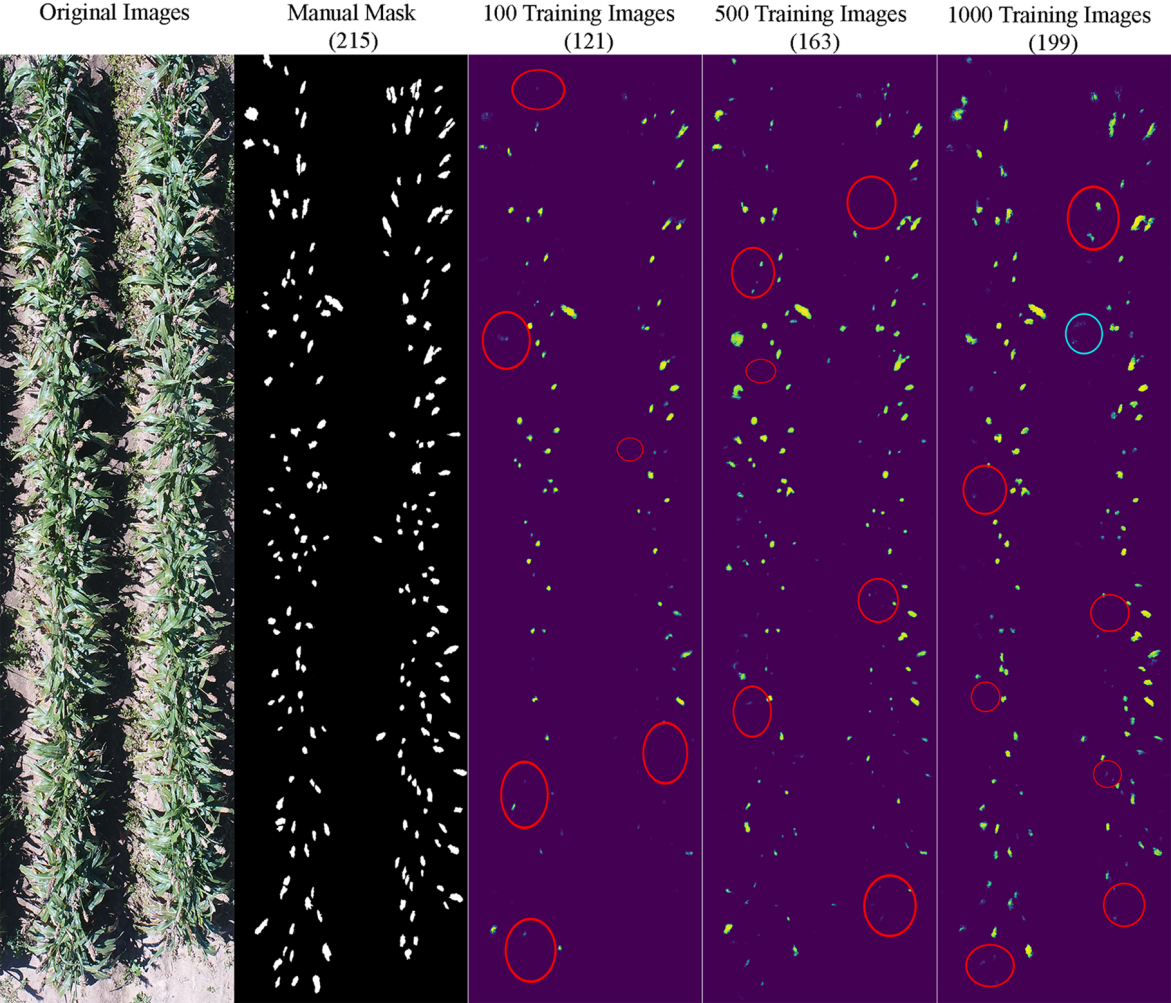
Sample results of sorghum panicle detection for Variety KS585 with 100, 500, and 1000 training images using a Convolutional Neural Networks model on UAS images. Red circles represent underestimation; blue circles represent overestimation compared to the manual masks.

**Figure 7:**
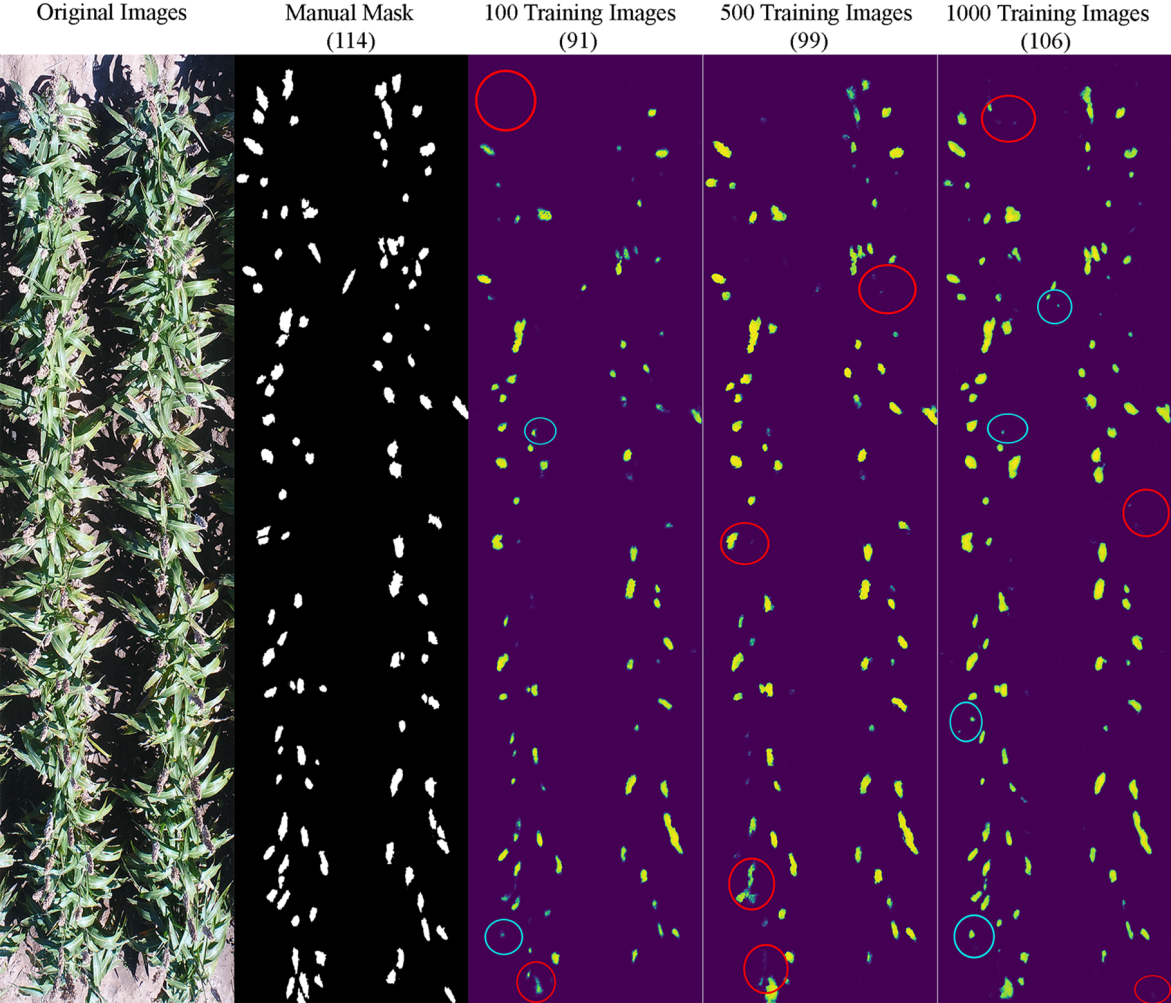
Sample results of sorghum panicle detection for Variety NK180 with 100, 500, and 1000 training images using a Convolutional Neural Networks model on UAS images. Red circles represent underestimation and blue circles represent overestimation compared to the manual masks.

For the case of 1,000 training images, sorghum panicles were overestimated for variety XM217 (168 predicted vs. 175 observed). For KS585, 199 sorghum panicles were predicted compared to 215 observed. For NK180, 106 panicles were predicted compared to 114 observed. The errors in these cases were mainly caused by overlapping sorghum panicles. For some images with bright soil background and leaves, the algorithm could not perfectly separate sorghum panicles from surroundings, which led to the underestimation errors. This situation was more widespread with a small number of training images, especially for sorghum varieties KS585 and NK180, which had bright panicles similar to the soil and shiny leaves.

## Discussion

Previous studies on sorghum panicle detection and counting used points or rectangular bounding boxes to label sorghum panicles for preparing the training datasets and outputting the predicted results (Guo et al., 2018; Ghosal et al., 2019; Oh et al., 2019). For example, Wu et al. (2019) combined the image segmentation technique and basic CNN algorithm to create a density map of sorghum panicles. The application of image segmentation can exclude the areas that are not directly involved in training dataset preparation and final output. Malambo et al. (2019) applied a semantic segmentation-based CNN algorithm to separate sorghum panicles from the soil and other parts in images. These machine learning algorithms for sorghum panicle detection were mainly based on image classification. The use of points or bounding boxes does not provide direct information about the sorghum panicle shape and size. Compared to previous similar studies, the U-Net CNN segmentation adopted in this study not only detect but also localize and delineate individual sorghum panicles. Therefore, the use of sorghum panicle masks and deep learning from this study enables the characterization of individual sorghum panicles, leading to more accurate yield estimation. This, however, does not mean sorghum yield can be directly calculated from the images because the sorghum panicles are typically not orthogonal to the UAS sensor during image acquisition. Further research is required to more accurately determine the size and shape of the sorghum panicle if yield prediction is needed.

**Figure 8:**
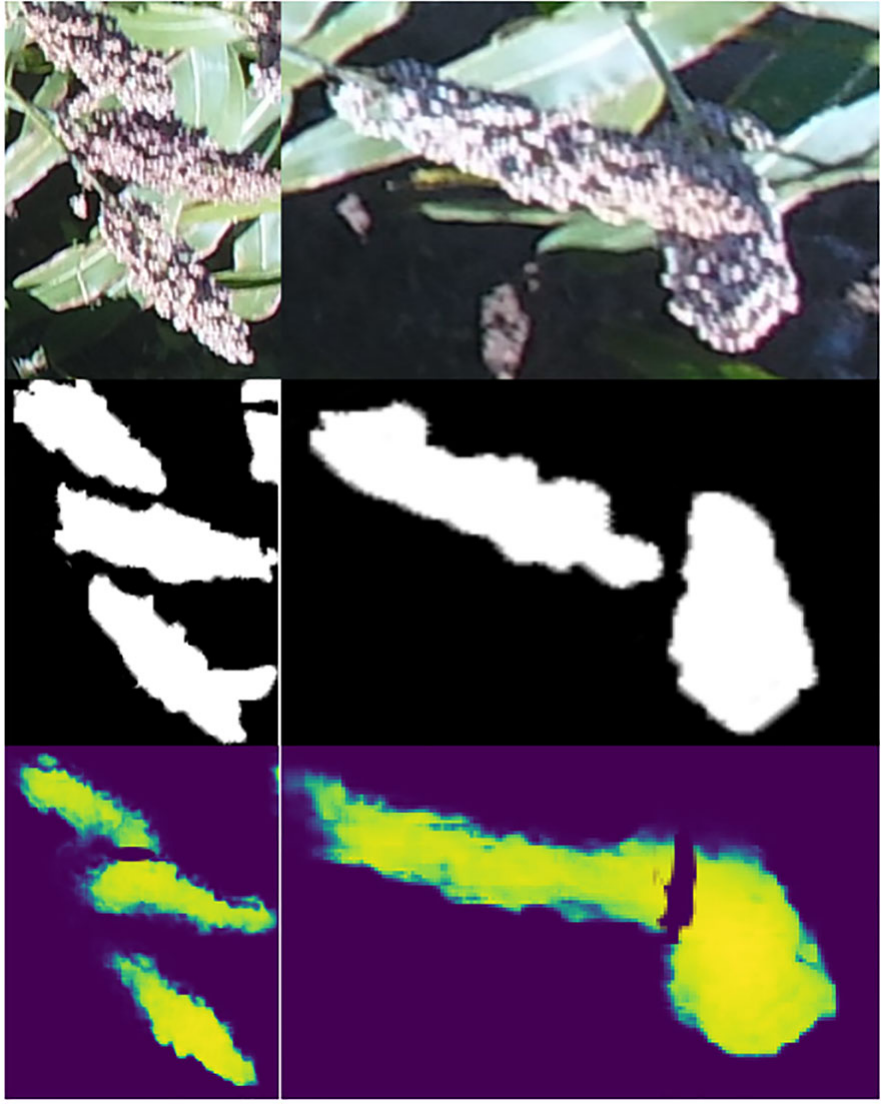
Sample images showing the minimization of errors in sorghum panicle detection due to overlaps using masks and deep learning. Upper images are raw images. Middle images are manually training masks. Bottom ones are predicted masks.

By using masks, our algorithm also minimized the errors in sorghum panicle detection due to panicle overlaps and mixing with other elements in the image. Agricultural images acquired using UAS typically have a mixture of target items and background elements due to the deformation caused by camera angel and other factors (Kamilaris and Prenafeta-Boldú, 2018; Pradeep et al., 2018). This makes object detection in computer vision tasks challenging, especially for the multiple overlapping panicles and panicles that are obscured partially by plant leaves (Guo et al., 2018). Chen et al. (2017a) used the DeepLabv3+ to detect object boundaries, with a high accuracy using 11,530 high quality pixel-level annotated images. However, this proposed algorithm was only able to separate the object boundaries between two different classes, but could not detect overlapping sorghum panicles described in this study. Similar methods all required a large number of well labeled training images. Compared with these methods, our algorithm was able to separate and count sorghum panicles individually. For example, **Figure 8** shows the overlapping panicles situation and the prediction results using training images and corresponding masks. As shown, the algorithm was able to detect overlapping sorghums by providing masks that mark overlapping panicles. This algorithm, however, could not detect all overlapping panicles due to the lack of training masks in such cases. We believe the performance in detecting overlapping panicles can be improved by increasing the number of overlapping training images.

One of the limitations encountered in this study was the split of a full image into pieces for counting sorghum panicles due to computation restrictions. Previous studies have also shown such challenges in machine learning and deep learning algorithms to directly process high-resolution images. It is common to crop or split the original large dimension images to smaller images for detecting and counting objects (Aich et al., 2018; Wu et al., 2019; Chandra et al., 2020). This potentially leads to overestimation. In our study, some sorghum panicles were cut into two parts and counted twice because we horizontally split the test image into four sub-images. However, our visual check indicated that most of panicles were not split evenly, resulting in the smaller pieces with less than six pixels not being counted. Therefore, the double counting issue had no significant effect on the accuracy of the algorithm. Future studies are required to address this limitation by adopting more efficient image processing algorithms to avoid potential double counting.

In this study, sorghum panicles with greater contrast in color and brightness with surrounding elements were easily detected and counted, while some other panicles, especially for the variety KS585, were challenging to detect due to their similarity to the surrounding features, including soil and dry leaves. Environmental factors, such as wind and clouds, have a significant impact on UAS image quality, which can affect the performance of deep learning algorithms. Field condition and plant genotypes also affect the accuracy of machine learning tasks (Torres-Sánchez et al., 2013; Rasmussen et al., 2019). The similar colors between soil background and crops could also cause errors in computer vision tasks (El-Faki et al., 2000; Lee et al., 2018). In this study, it appears some soil clusters and leaves were mislabeled as sorghum panicles, probably due to strong sunlight conditions. We acquired most of the images around local noon time. As a result, both the soil surface and some sorghum panicles were relatively bright in full sunlight. In future studies, users may consider acquiring UAS images under relatively soft light environments, such as late afternoon or early morning. For the improvement of the algorithm performance, adding a separate mask for soil pixels can be an effective alternative to separate the sorghum panicles from soil background.

Abundant training datasets are critical for effective deep learning tasks (Deng et al., 2014; LeCun et al., 2015), especially for complex computer vision tasks such as sorghum panicle detection and counting. This study provides useful information regarding the number of training images required for such deep learning tasks. The algorithm produces inconsistent predictions and low accuracy with below 500 training images. It is reasonably accurate with 1,000 training images. It is expected that with more training images, the accuracy and robustness can be further improved. Aggarwal et al. (2019) demonstrated that a large number of training images could improve the performance of the U-Net CNN model, especially in complex models. However, there are not enough public ready-to-use data as training datasets for specific crops and their phenotypic traits. The development of large training datasets for plant phenotyping is time-consuming and labor-intensive. The drawback of the pre-label based algorithm lies in the fact that it is time consuming to prepare these training masks of sorghum panicles. In this study, 1,000 training images and masks were manually prepared and applied to develop the algorithm. It took a considerably longer time to prepare the training datasets compared with previous studies that used dot-labeled training images. The automatic annotation technique has shown its potential in similar algorithms (Zahavy et al., 2016; Komura and Ishikawa, 2018; Ghosal et al., 2019). Predicted outputs from automatic annotation can be used as new training input, which reduces the workload of manual preparation and can improve the efficiency and the robustness of the algorithm.

## Conclusions

In this study, we developed an algorithm to integrate deep learning and segmentation to detect and count sorghum panicles using high-resolution UAS images. A dataset of 1,000 randomly selected images and their corresponding manually labeled masks were constructed for training this algorithm. The performance and efficacy of the algorithm were assessed with a different number of subset training images. The performance of the algorithm improved with the number of training images. The performance of the algorithm was not stable with less than 500 training images. With 1,000 training images, the algorithm had the best performance, with an accuracy of 95.5% and an RMSE of 2.5. The algorithm is sufficiently accurate for varying orientations and sizes of three sorghum varieties. Therefore, future studies are required to test the robustness of our algorithm with other varieties. In addition, compared to previous similar studies, our algorithm integrated image segmentation and CNN deep learning, which not only detect but also localize and delineate individual sorghum panicles. The algorithm is also capable of detecting overlapping sorghum panicles. This offers an opportunity for enhanced sorghum breeding efficiency and accurate yield estimation. To achieve this, however, further research is needed to improve the algorithm to quantify panicle dimension in relation to yield.

The development of large training datasets for plant phenotyping is time consuming and labor intensive. Therefore, this study provides a benchmark for the requirement for the number of training images for such phenotyping tasks. On the other hand, a more effective method, such as automatic annotation, is needed to prepare reliable training images. The performance of this algorithm was evaluated at the small-plot scale. Further studies are required to expand this algorithm to detect and count sorghum panicles at the commercial field scale. In addition, sorghum panicle detection accuracy as influenced by environmental factors, including image resolution, soil background, and illumination levels, requires further evaluation.

## Data Availability Statement

The datasets generated for this study are available on request to the corresponding author.

## Author Contributions

Conceptualization: WG and ZL. Methodology: ZL and WG. Software: ZL. Validation: ZL and WG. Formal analysis: ZL and WG. Investigation: ZL and WG. Resources: WG. Data curation: ZL. Writing—original draft preparation: ZL. Writing—review and editing: ZL and WG. Visualization: ZL and WG. Supervision: WG. Project administration: WG. Funding acquisition: WG.

## Conflict of Interest

The authors declare that the research was conducted in the absence of any commercial or financial relationships that could be construed as a potential conflict of interest.
